# Does early sexual debut reduce teenagers' participation in tertiary education? Evidence from the SHARE longitudinal study

**DOI:** 10.1016/j.adolescence.2009.10.006

**Published:** 2010-10

**Authors:** Alison Parkes, Daniel Wight, Marion Henderson, Patrick West

**Affiliations:** Medical Research Council Social and Public Health Sciences Unit, University of Glasgow, 4, Lilybank Gardens, Glasgow, G12 8RZ, United Kingdom

**Keywords:** Sexual debut, Education, Longitudinal, Teenager, Pregnancy

## Abstract

Negative effects of early sexual debut on academic outcomes can extend beyond secondary school, although concurrent changes in other psychosocial risk factors have not been investigated. Data from three waves of a longitudinal survey of Scottish teenagers were used to examine associations between early sexual debut (first heterosexual intercourse) and both expectations for (*N* = 5,061) and participation in (*N* = 2,130) tertiary education at college or university. Early debut was associated with reduced tertiary education, after adjusting for academic performance and wave 1 confounders relating to social background, attitudes and behaviours. Pregnancy/partner pregnancy did not explain all of this finding, as many sexually experienced teenagers opted out of tertiary education after leaving school early for other reasons. Changes in other psychosocial risk factors between waves 1 and 2 mediated much of the association found. Early sexual experience may predict disengagement from tertiary education, although further research is needed to explore causal pathways.

## Introduction

Low academic achievement and aspirations have been widely identified as risk factors for early sexual debut in longitudinal studies (Zimmer-Gembeck & Helfand, 2008). There has been less research on associations between teenage sexual debut and subsequent academic underachievement. Such associations may not involve causal effects: sexual debut and a decline in academic aspirations and performance during adolescence may lie on the same developmental trajectory, with shared antecedent risk factors. Other theoretical models suggest direct effects of sexual debut. Specific effects include the possibility of disruption to education caused by pregnancy or sexually transmitted infections, and less time and concentration on educational objectives if teenagers are pre-occupied with sexual activity and dating (Safron, Schulenberg, & Bachman, 2001). Sexual debut may also lead to emotional problems, such as depression and low self-esteem (Meier, 2007), which could impede school work (Grimm, 2007).

More generally, the effects of early sexual activity may resemble those of other risk behaviours, viewed collectively as a ‘problem behaviour syndrome’ (Jessor, 1991). Engaging in risk behaviours is thought to mark ‘transition proneness’: early adoption of adult roles (Jessor & Jessor, 1975; Udry & Billy, 1987). Early sexual activity within a romantic relationship could increase expectations of cohabitation, marriage and childbearing (Manning, Longmore, & Giordano, 2004; Marini, 1985). Early sexual activity and other risk behaviours often accompany adolescent employment (Bachman, Safron, Sy, & Schulenberg, 2003; Bozick, 2006), a transitional role that might tempt an adolescent to forgo investment in tertiary education (Marini, 1985). Interactional theories of development (Thornberry, 1987) suggest a causal basis between involvement in risk behaviours and the development of a less conventional outlook. Engagement in risk behaviours antagonizes relations with conventional supports such as family and school, and strengthens ties with unconventional peer groups (Jessor & Jessor, 1975). This model is supported by longitudinal research linking sexual debut with subsequent changes over time, including poorer relationships with parents, lower religious attendance, stronger affiliation with deviant peers and increased likelihood of delinquency (Armour & Haynie, 2007; Billy, Landale, Grady, & Zimmerle, 1988; Ream, 2006; Ream & Savin-Williams, 2005). Such changes might foster a shift in a teenager's mindset, with less value placed on education. Effects of sexual debut on diminished school affiliation (Billy et al., 1988; Ream, 2006) could lead more directly to lower academic performance and aspirations. A similar model has been suggested to explain longitudinal associations between adolescent substance use and depressed academic outcomes (Fergusson, Horwood, & Beautrais, 2003; Georgiades & Boyle, 2007; King, Meehan, Trim, & Chassin, 2006; Lynskey & Hall, 2000; Lynskey, Coffey, Degenhardt, Carlin, & Patton, 2003; Macleod et al., 2004; Mathers, Toumbourou, Catalano, Williams, & Patton, 2006).

There is some evidence that early sexual debut has negative associations with academic outcomes at secondary school that are not explained by prior confounding influences. A few studies found longitudinal associations between sexual debut and depressed academic aspirations and/or achievement in US secondary school pupils, after taking account of initial levels of these academic outcomes (Billy et al., 1988; Ohannessian & Crockett, 1993; Schvaneveldt, Miller, Berry, & Lee, 2001). These studies looked at effects of sexual debut over periods of two to five years, and adjusted for the effects of family background. More recently, negative effects of sexual debut on school attachment and performance were found after adjusting for a much wider range of prior confounders, as well as for changes in some of these over the 12-month period studied (Sabia, 2007a, 2007b).

Recent US research has also investigated effects on education persisting beyond secondary school. Sexual debut before age 16 was associated with decreased early adulthood participation in tertiary education, after adjusting for confounders measured 6–7 years previously. These included academic achievement, family and neighbourhood factors, college expectations and aspirations, and substance use (Spriggs & Halpern, 2008). The study suggested that childbearing accounted for much of the negative association between sexual debut and educational participation, but did not explore the contribution of changes in teenagers' social environment and attitudes over the study period.

The current paper extends this research on longitudinal associations between sexual debut and tertiary education to a UK setting. In addition to non-participation in tertiary education, it examines earlier expectations for such education; and undertakes an exploration of psychosocial changes associated with sexual debut. Theoretical considerations suggest that early timing of debut is important, reinforced by a recent review suggesting different developmental pathways associated with debut during early, middle and late adolescence (Zimmer-Gembeck & Helfand, 2008). Sexual debut in the late teens reflects both normative timing and fewer concurrent psychosocial risk factors, and so we focused on debut either by mean ages 14 or between mean ages 14 and 16 years, similar to divisions used in recent US research (Sabia, 2007a, 2007b; Spriggs & Halpern, 2008). We test for gender differences in the effects of debut, found in previous research (Billy et al., 1988; Ohannessian & Crockett, 1993; Sabia, 2007b; Schvaneveldt et al., 2001; Spriggs & Halpern, 2008).

## Method

### Data set

Following approval by Glasgow University's Ethical Committee for Non-Clinical Research Involving Human Subjects, twenty-five schools in eastern Scotland, UK participated during 1996–2002 in a randomised control trial of enhanced school-based sex education, SHARE (Wight et al., 2002). All third-year pupils in two successive cohorts were invited to take part (*N* = 8430). This study uses data from three waves spaced at two-yearly intervals (at mean ages 14, 16 and 18 years). Respondents provided information in a self-complete, anonymised questionnaire. At waves 1 and 2 this was administered in school by researchers in examination conditions, except for postal questionnaires for those who had left school by wave 2. At wave 3, all were sent postal questionnaires, but had the option of responding to the same questionnaires by telephone or internet. Only 283 respondents completed web-based or telephone questionnaires.

The analysis combined both arms of the trial, which found no difference in sexual behaviour self-reported at wave 2 and no difference in linked health service data on conceptions or terminations by age 20 (Henderson et al., 2007; Wight et al., 2002). As a precaution, all the analysis adjusted for arm of trial (all effects NS *p* < 0.05).

### Analysis samples and weighting

5356 pupils filled in questionnaires at both waves 1 (total *N* = 7616) and 2 (total *N* = 5854), but 196 were given a shorter postal questionnaire at wave 2 that was not suitable for our purposes. This left 5160 pupils eligible for analysis at wave 2. After removing cases with missing outcome information, there were 5061 cases (2359 boys and 2702 girls) included in analysis.

At wave 3, out of 2855 teenagers who returned a questionnaire 2174 were eligible for analysis (responding at all three waves). After removing cases with missing outcome information, there were 2130 cases (724 boys and 1406 girls) left for analysis.

At wave 1, the sample was representative of the Scottish population, in terms of parental social class and family structure (Wight et al., 2002). The less convenient postal rather than school classroom administration of the questionnaire was the main reason for the decreased response rate among early school leavers at wave 2 and all wave 3 teenagers, despite the use of different completion methods, reminders and incentives to maximize wave 3 response.^1^ Incorrect addresses accounted for under 10% of non-responses at wave 3.

To compensate for differential attrition (greater for boys and high risk groups), multivariate models predicting wave response were developed, using backward conditional regression on measures found to have significant (*p* < 0.01) univariate associations with response. This regression included all variables at the start, with removal testing using the probability of the likelihood-ratio statistic based on conditional parameter estimates. Inverse values of predicted response probabilities were used to weight cases. The use of weights increases sample variance, increasing the risk of type 1 error. To help counteract this effect, the top 5% of weights were trimmed down to the value corresponding to the maximum attained by 95% of cases (Höfler, Pfister, Lieb, & Wittchen, 2005). Important wave 1 predictors of wave 2 response included gender, cohort, school response rate, parental social class, housing, home postcode deprivation, school postcode deprivation, level of school placing requests,^2^ self-reported honesty of completing the questionnaire, future expectations, truancy and the proportion of friends who had left school. Wave 2 predictors of wave 3 response included gender, reward for study participation, school grades, home postcode deprivation^3^ and self-reported honesty of of the questionnaire completion.

### Outcomes

#### No expectation of tertiary education

At wave 2, pupils were asked whether they expected to be at college or university in two years' time. Responses on a 5-point Likert scale were converted to a binary measure contrasting ‘unsure/unlikely/very unlikely’ with ‘very likely/likely’. The 5-point measure was negatively correlated with expectations for employment (−0.40, *p* < 0.01). However, as many students finance their studies with paid employment, we cannot use employment expectation as a robust alternative measure of educational expectation.

#### No participation in tertiary education

All were asked at wave 3 what they were doing in the current academic year, choosing one or more of five main options covering education and employment, or describing other activities (coded for educational content). A variable was created contrasting non-participation with engagement in some form of tertiary education. The participating group included those who said they were deferring tertiary education for a ‘gap year’.

#### Key independent variable: sexual debut

Sexual debut was defined as first vaginal intercourse, ‘a boy/man putting his penis into a girl/woman's vagina’ or ‘going the whole way’. The timing of debut was classed as either ‘wave 1’ (debut reported by wave 1) or ‘wave 2’ (debut reported by wave 2, but not at wave 1). Teenagers reporting wave 1 debut were only included provided they confirmed debut at wave 2: this procedure was supported by increased self-reported honesty of questionnaire completion with age, in line with other studies (Siegel, Aten, & Roghmann, 1998; Spriggs & Halpern, 2008).

### Analysis and further independent variables

Logistic regression modelled outcomes using MLwiN version 2.0, which allowed for clustering by school and weighting. Dummies were included for missing categories of independent variables. All models adjusted for age at wave 2, gender and arm of trial.

The first modelling stage explored whether there was any effect of sexual debut (wave 1 and/or wave 2) on the two tertiary education outcomes, after taking account of academic achievement at wave 2. Academic achievement was measured using self-reports of Standard Grades, Scotland's educational qualifications taken by secondary school pupils aged 15–16 years (broadly equivalent to the General Certificate of Secondary Education qualification taken in other parts of the UK). Scores were created from the best five Standard Grades passed, awarding 6 points for a band 1 (highest level of achievement), 5 points for a band 2 and so on down to band 6 (the minimum pass level). Scores were banded into deciles, combining the two top deciles due to the large number of top scores.

The second modelling stage explored whether any effect of sexual debut by wave 2 could be explained by prior influences: social background and wave 1 school engagement, expectations and psychosocial factors. All these factors have been associated with both sexual debut and academic outcomes in the research literature (confirmed by strong (*p* < 0.001) univariate associations in our data set). Measures of social background comprised a six-fold measure of highest parental social class,^4^ highest parental educational level (six-part classification, from a degree/advanced qualification to ‘left school at 16’), whether the teenager lived with both biological parents, neighbourhood deprivation and school-level percentage of pupils retained past the minimum school leaving age.^5^

School engagement was measured by agreement with statements ‘I like school’ and ‘When I get the chance I skip school’ (5-point scales). Expectations for four years' time comprised expectations of college/university, employment, cohabiting with a partner and having a child (5-point scales).

Two psychosocial measures, average weekly spending money and current boy/girlfriend, were included as likely precursors to adult transition. Although some money might come from parents, larger sums are a strong indicator of paid employment outside the home (West, Sweeting, Young, & Robins, 2006). Current relationship also distinguishes the effects of sexual debut from that of romantic attachments (Ream, 2006; Sabia, 2007a, 2007b).

The remaining set of psychosocial factors include measures of social ties, self-esteem and risk behaviours that may be good indicators of unconventionality (Jessor, 1991). Parental monitoring and self-esteem each consisted of mean response to four questions (respectively, rules for going out in the evening, alpha = 0.67; and items from a shortened version of the Rosenberg Self-Esteem scale, alpha = 0.62). Friends' composition was a response to the question ‘How many of your friends have left school?’ (1 = ‘none’ to 5 = ‘all’) and religiosity a 4-point scale (‘religious’ to ‘not at all religious’). Cigarettes, alcohol and cannabis were the three substances most often used by teenagers in the survey. Cigarette smoking and cannabis use were measured using 4-point scales (‘never tried’, ‘tried’, ‘use occasionally’, ‘use regularly’). Alcohol use was measured a 5-point drunkenness scale (‘never drunk’, and drunk ‘once or twice a year’, ‘about once a month’, ‘about once a week’ or ‘more than once a week’). This measure was found in a pilot to correspond closely with amount of alcohol consumed (*r* = 0.67, *p* < 0.000).

In the third modelling stage, we explored whether particular events and/or *changes* in expectations and psychosocial factors over time provided any further explanation for the effects of sexual debut. The two events included were pregnancy/partner pregnancy and early school leaving. We used wave 2 information on these events for the model of wave 2 tertiary education expectations, and wave 3 information for the model of wave 3 tertiary participation. Information on pregnancy/partner pregnancy collected at wave 2 was missing from teenagers attending nine schools in one education authority that did not permit sensitive questions. A dummy variable was included for this authority. Wave 3 reporting of pregnancy/partner pregnancy was collected from all respondents, regardless of education authority. From this information we derived a measure of pregnancy/partner pregnancy occurring between waves 2 and 3, for those who reported wave 2 sexual debut. Information on early school leaver status was collected by schools prior to the wave 2 survey. A three-way variable was created: (1) still at school, older than minimum school leaving age (2) still at school but not yet attained minimum leaving age (3) early school leaver. By wave 3, all respondents had left secondary school and reported on when they had done so. Changes in expectations and psychosocial measures over time were modelled at stage 3 by including wave 2 values corresponding to wave 1 measures already included at stage 2 (for example, wave 2 expectations of employment, spending money and parental monitoring).

## Results

Selected characteristics of the samples at all three waves are shown in Table 1. The wave 2 and 3 figures are weighted values. When sample characteristics available at all three waves are compared, it can be seen that between-wave differences are small: this indicates that weighting has in general succeeded in restoring the wave 2 and 3 samples to the baseline composition. The difference in ‘wave 1 sexual debut’ between wave 1 and later samples reflects the decision to use wave 1 debut only if information was confirmed at wave 2 (see Methods). Weighting was least effective for boys in the wave 3 sample, who were under-represented at this time point. For instance, wave 3 boys contained slightly higher percentages of those in two-parent families, from professional/managerial classes and more affluent neighbourhoods than at waves 1 and 2.

Table 1 shows that at wave 2, about three in ten teenagers did not expect to be in tertiary education in two years' time, with more boys than girls expressing this belief (*p* < 0.001). By wave 3 four in ten teenagers were not in tertiary education. There was no gender difference in participation, although fewer boys than girls had completed all six years of secondary school education (*p* < 0.01).

A minority of teenagers (15%) reported sexual debut at wave 1, although a further 28% reported debut between waves 1 and 2. Sexual debut was associated with lower expectations for tertiary education at wave 2: 45% of wave 1 and 39% of wave 2 debut groups did not expect to participate, compared to only 24% of virgins at wave 2. A similar effect was found for non-participation in tertiary education: 63% of wave 1 and 50% of wave 2 debut groups did not participate, compared to only 29% of wave 2 virgins. Sexually experienced teenagers were exposed to the risk of pregnancy or partner pregnancy, which in turn was associated with poorer educational outcomes (Table 2, which shows univariate associations). Both sexually experienced teenagers and those not expecting/participating in tertiary education were less likely to complete their secondary school education. In addition, Table 2 shows that many attitudes and risk behaviours were common to both the sexual and educational risk groups. For ease of presentation in this Table, scale measures have been converted to binary scores. Patterns were most obvious at wave 1, but sexual debut and educational outcomes were also associated with new reporting of many attitudes and risk behaviours at wave 2.

Stage 1 of multivariate modelling shows the effect of sexual debut on educational outcomes, adjusting for wave 2 academic achievement (Table 3, first set of columns for each outcome). Although achievement was strongly associated with both outcomes, there was a significant effect of sexual debut at both waves. Note the formal difficulty in comparing expectations and participation models, as the latter uses a sub-sample of the first model. The expectations model was repeated using the wave 3 sub-sample and gave similar results, but the full wave 2 analysis using a larger, more representative sample is shown here. A test of the gender × sexual debut interaction found no significant gender difference in the effect of sexual debut on either outcome. At stage 2, the effect of sexual debut was partially reduced with the addition of social background and wave 1 measures of school engagement, expectations and psychosocial factors (Table 3, second set of columns for each outcome). In both models the adjustment effect was stronger for wave 1 than for wave 2 sexual debut.

The third stage of multivariate modelling involved adjusting for events (pregnancy and early school leaving); and for changes in expectations and psychosocial factors between waves 1 and 2. The effects of sexual debut on tertiary education outcomes after different sets of adjustments in the models are shown in Table 4. Adjusting for pregnancy (Table 4, Model 1) resulted in only a small additional effect on the odds attributed to wave 2 debut in both models, although there was a larger decrease in the odds attributed to wave 1 debut in the participation model. Teenagers were asked to provide extra information on what had happened following a pregnancy, and their responses were divided into those reporting a miscarriage or termination and those who had experienced or anticipated a birth. However, the effect of sexual debut on tertiary education was unaffected by this extra information on pregnancy outcome (not shown). There was no gender difference in the effects of pregnancy, or of a termination/miscarriage compared to a birth.

Model 2 in Table 4 shows the effect of adjusting for early school leaving as well as pregnancy. In the expectations model, leaving school by wave 2 produced a slight further decrease in the odds attributed to wave 2 sexual debut. Further examination of the timing of wave 2 sexual debut in relation to school leaving supported the notion that sexual debut generally preceded, rather than followed leaving at this wave. ‘Early leaving’ in the participation model comprised all leaving before the end of six years of secondary school, and attenuated the odds attributed to sexual debut to non-significance.

Models 3–6 in Table 4 show the effect of adding additional wave 2 expectations and psychosocial measures on the odds attributed to sexual debut. All models adjusted for pregnancy but not early school leaving, so they should be compared with Model 1. Since models already included corresponding wave 1 measures, this allows exploration of *changes* that might explain why sexually active teenagers were less likely to take up tertiary education. Model 3 shows that changes in ‘transition expectations’ of job, cohabitation and childbearing reduced some of the effects of wave 2 sexual debut. When wave 2 educational expectations were added to the participation model (model 3a), much of the remaining effect of sexual debut was accounted for.

Models 4–6 show the effect of sexual debut on education after taking account of changes in various psychosocial factors between waves 1 and 2. For both outcomes, changes in current relationship and spending money (Model 4) and in psychosocial factors associated with greater unconventionality (Model 5) reduced the odds attributed to wave 2 sexual debut, with Model 6 (both sets of factors) producing the greatest adjustment. However, only changes in current relationship and spending money attenuated the effect of wave 2 sexual debut on participation to non-significance. Wave 1 sexual debut remained a significant predictor of lower tertiary participation in Models 4–6. Adding the changes in transition expectations to Model 6 did not result in further adjustment of the effect of sexual debut on participation (not shown).

## Discussion

This UK study found that sexual debut by age 16 was associated with reduced expectations for and participation in tertiary education, regardless of the level of academic achievement at age 16. Differences in family, neighbourhood and school background; together with differences in school engagement, expectations and psychosocial factors measured two years' previously only partially accounted for these effects. The remaining significant negative effects of early sexual debut on tertiary education confirm the findings of a recent US study (Spriggs & Halpern, 2008).

A similar effect of sexual debut was found in both girls and boys, unlike previous US research which has found evidence of gender differences (Billy et al., 1988; Ohannessian & Crockett, 1993; Sabia, 2007b; Schvaneveldt et al., 2001; Spriggs & Halpern, 2008); and it was similar for wave 1 (by age 14) and wave 2 debut (between the ages of 14 and 16), in agreement with US research (Sabia, 2007a, 2007b; Spriggs & Halpern, 2008). Our predominantly white sample precluded investigation of ethnic variation in the effects of early debut.

Pregnancy/partner pregnancy did not explain all of this relationship, and it appears that many sexually experienced teenagers opted out of tertiary education after leaving school early for reasons other than pregnancy. Indeed pregnancy may have followed, rather than precipitated, school leaving. Unfortunately we only have partial information on whether pregnancy occurred before, or after, school leaving. A New Zealand study that found associations between pregnancy and reduced academic achievement noted that in the majority of cases, pregnancy occurred after leaving school (Fergusson & Woodward, 2000). Girls who became pregnant while at school were more likely to terminate their pregnancy and remain in school, with less disruption to their longer-term education (Fergusson, Boden, & Horwood, 2007). Our results differ from the recent US study (Spriggs & Halpern, 2008) on non-initiation of post-secondary education, which found that childbearing ‘explained’ most of the effects of early sexual debut. This might reflect cultural differences, as well as the much older age range of the final wave in the US study compared to our own. Our study examined participation in tertiary education immediately after respondents had left school: in the US study, many participants were several years out of secondary school.

The association between early school leaving and non-participation in tertiary education is not as automatic as it may first appear. Access to some tertiary courses, particularly those leading to a university degree, depends on the qualifications achieved after completing five or six years of secondary school education in Scotland. In addition, there are tertiary education courses that accommodate teenagers without such qualifications. Thus teenagers who leave school at or soon after the minimum age for compulsory secondary education (normally around the time of their sixteenth birthday) are still able to participate in some form of tertiary education. We did not model school leaving as a main outcome, as wave 2 measures (including academic achievement) were not collected until after the minimum school leaving age for many pupils. However our findings suggest that sexual debut leads to lowered academic aspirations and subsequent school drop out. At least in the short term, these teenagers are less likely to re-enter education at the tertiary level, regardless of academic achievement at age 16. Our results echo previous research suggesting links between substance use and school leaving (Ellickson, Bui, Bell, & McGuigan, 1998; Ellickson, Tucker, & Klein, 2001; Fergusson et al., 2003; Lynskey et al., 2003; Newcomb et al., 2002).

Sexual debut was accompanied by greater spending money (probably indicating that a teenager has a paid job outside the home) and greater likelihood of having a boy/girlfriend, which may both act as precursors to early transition to adulthood – in keeping with this view, we found that increased expectations of early transition to adult roles of employment, cohabitation and childbearing helped to account for the effects of debut, even when experience of pregnancy was allowed for. Other changes (lowered parental monitoring and religiosity, together with having more friends who had left school and more frequent engagement in other risk behaviours) are likely to be associated with less ‘conventionality’ (including a lower value placed on education). The greatest reduction in the effect of wave 2 sexual debut on tertiary education was produced by taking account of changes in both transitional precursors and conventionality. The study echoes research on academic performance and motivation in US secondary school pupils, which found attenuated effects of sexual debut after taking account of time-varying confounders (Sabia, 2007a, 2007b). It extends US work on initiation of post-secondary education beyond the incorporation of baseline confounders and information on childbearing (Spriggs & Halpern, 2008). However, the causal framework underlying these effects is unclear. Early sexual debut may precipitate social and motivational changes, the reverse effect may operate, or sexual debut and psychosocial changes may lie on common developmental trajectories with no direct causal linkages.

Limitations of the study include the validity of self-reported data on academic achievement and sensitive behaviours (Brener, Billy, & Grady, 2003), reliance on many single-item measures, and a risk of bias due to issues surrounding missing data. However, data collection was designed to preserve anonymity and respect confidentiality, and levels of achievement, school leaving rates and risk behaviours were comparable with self-reports in national surveys of Scottish teenagers (Currie, Levin, & Todd, 2008; Lynn, Nicolaas, & Pitson, 2000). Weighting cases in the analysis helped to counteract the effects of differential attrition, restoring wave 2 and 3 samples to the representative wave 1 social composition. The results are thereby more likely to be generalisable to other similar populations of teenagers, although even with the use of weights there were small compositional differences between wave 3 and earlier samples for boys. A second issue with missing data is non-response for questionnaire items. List-wise exclusion of cases with missing outcome information was at low levels (2% in both waves). Missing information for independent variables was also generally at low levels (well below 10% for the standard controls, with the exception of parental education at 13% in wave 2 and 12% in wave 3). Those with missing parental education information did not differ from the rest in terms of sexual debut. Levels of missingness in the independent variables were not great enough to warrant multiple imputation (Widaman, 2006). A greater amount of pregnancy information was missing at wave 2 from one local education authority: however at wave 3 only 2% of cases had missing pregnancy information.

The analysis presented here focused on first heterosexual intercourse, and did not consider other aspects of sexual behaviour. We were limited by partial data collection for oral sex (none at wave 1 and missing from one education authority at wave 2), but available information on a range of sexual behaviours (kissing, touching genitals and oral sex) suggested that only intercourse was significantly associated with decreased expectations of tertiary education. Exploratory analysis suggested no clear differences in expectations according to intercourse frequency, number of partners or condom use; in line with other findings of the effects of sexual debut on school attachment (Sabia, 2007a). Other research has suggested lower school attachment for those combining sexual activity with other risk behaviours, compared to those with sexual activity only (Ensminger, 1990), and more negative adult outcomes for teenagers engaging in a greater number of risk behaviours (Viner, 2005). However further exploration of our data did not suggest significant interactive effects of sexual debut with substance use measures.

A further limitation is the lack of some potential confounders that are available for similar analyses using richer data sets, such as measures capturing parental support for education, quality of parent-child relationship, family functioning, parental adjustment, early individual conduct and attentional problems, delinquency and measures of physical and mental health (Fergusson et al., 2003; Georgiades & Boyle, 2007; Sabia, 2007a, 2007b). Cultural influences are another potential unmeasured confounder, although a lack of significant between-school variation in educational outcomes at the second stage of our multilevel models suggests that we may have already captured these in our controls for family, school and neighbourhood background. Although our range of baseline adjustments is comparable to that offered by the related US study on sexual debut and tertiary education (Spriggs & Halpern, 2008), a better understanding of confounders could erode some of the effect attributable to sexual debut in this study. On the other hand, because examination grades were measured at one time point only we were not able to investigate whether sexual debut was negatively associated with academic performance, as shown in previous studies (Billy et al., 1988; Sabia, 2007b; Schvaneveldt et al., 2001). If sexual debut depressed educational expectations via reduced academic performance, we may have under-estimated its effects.

Unravelling the psychosocial bases and consequences of engagement in risk behaviours presents considerable methodological challenges for future research, which has begun to be addressed in longitudinal research on peer influences (Altermatt & Pomerantz, 2005; Kindermann, 2007). Even though underlying mechanisms require clarification, our results suggest that educators, policy makers and health professionals should take note of signals offered by early sexual debut for future academic achievement, and explore ways to increase risk-taking teenagers' engagement with school education.

## Figures and Tables

**Table 1:** Selected characteristics of teenagers in the SHARE study at waves 1, 2 and 3.

N values are unweighted.		Wave 1			Wave 2 eligible sample			Wave 3 eligible sample		
		Male	Female	All	Male	Female	All	Male	Female	All
		*3822*	*3794*	*7616*	*2415*	*2745*	*5160*	*745*	*1429*	*2174*
Gender (%)	Female			50			50			50
Ethnic group (%)	Non-white	4	4	4	5	4	4	4	4	4

*Family background*										

Highest parental social class (%)	Professional	8	8	8	8	8	8	9	9	9
	Managerial/Technical	36	36	36	36	34	35	39	35	37
	Skilled non-manual	25	25	25	25	25	25	24	25	25
	Skilled manual	19	18	18	18	18	18	17	19	18
	Partly skilled	9	10	9	8	10	9	8	9	9
	Unskilled	4	4	4	4	4	4	3	4	4

Live with both biological parents (%)		71	68	69	71	66	68	74	68	71

Neighbourhood deprivation category (%)	1 (most affluent)	13	13	13	13	12	13	15	12	14
	2	16	17	17	16	17	16	19	16	18
	3	20	18	19	20	18	19	19	20	19
	4	24	24	24	23	25	24	23	23	23
	5	14	15	15	14	16	15	11	17	14
	6	8	9	9	9	9	9	8	8	8
	7 (most deprived)	5	4	4	4	4	4	3	4	4

Highest parent educational level (information not collected at Wave 1) (%)	Degree/advanced qualification				48	45	46	51	45	47
	Attended college/university				24	23	24	23	23	23
	Highers				6	6	6	7	5	6
	Standard grades				12	13	13	12	14	13
	At school post-16				2	1	2	1	1	1
	Left school at 16				8	12	10	7	12	9

Wave 2 academic achievement	Mean grade band score				4.6	5.0	4.8	4.6	5.0	4.8

Leaving school early (%)	Reported at Wave 2 (close to minimum school leaving age)				14	19	17	15	20	17
	Before end of secondary school final year (S6)							55	49	52

Tertiary education (% participation)	Wave 3							58	62	60

*Expectations*										

Expect tertiary education (% likely/very likely)	Wave 1	59	71	65	59	69	64	63	69	66
	Wave 2				64	73	68	67	72	70

Expect job (% likely/very likely)	Wave 1	47	36	41	48	37	42	43	35	39
	Wave 2				40	37	38	36	34	35

Expect cohabit (% likely/very likely)	Wave 1	36	22	29	37	23	30	34	21	27
	Wave 2				15	15	15	14	15	14

Expect child (% likely/very likely)	Wave 1	15	11	13	15	11	13	11	11	11
	Wave 2				3	5	4	2	5	3

*Risk behaviours*										

Sexual debut (%)	Wave 1	18	15	17	14	17	15	10	15	12
	Wave 2				25	31	28	25	30	28

Pregnancy/partner pregnancy (%)	Wave 2				3	6	4	2	5	3
	Wave 3^a^							6	11	9

Truancy (% Strongly agree/agree skip school)	Wave 1	20	15	18	20	17	18	16	15	16

Cigarettes (% regular use)	Wave 1	11	17	14	10	19	15	8	17	13
	Wave 2				19	32	25	20	29	25

Alcohol (% drunk once a week or more)	Wave 1	17	19	18	15	21	18	9	20	15
	Wave 2				32	31	31	29	30	29

Cannabis (% regular use)	Wave 1	5	2	4	5	3	4	5	2	3
	Wave 2				10	5	7	8	4	6

*Other psychosocial factors*										

Current boy/girlfriend (%)	Wave 1	25	26	25	23	28	26	21	27	24
	Wave 2				26	40	33	28	36	33

Spending money (mean £ per week)	Wave 1	11.3	9.4	10.4	11.2	9.3	10.3	10.6	9.2	9.8
	Wave 2				23.6	24.3	24.0	21.9	24.0	23.1

Parental monitoring (mean score)	Wave 1	2.3	2.1	2.2	2.3	2.1	2.2	2.2	2.1	2.1
	Wave 2				2.6	2.4	2.5	2.6	2.4	2.5

Self-esteem (mean score)	Wave 1	2.1	2.4	2.2	1.9	2.2	2.0	2.0	2.3	2.1
	Wave 2				1.9	2.3	2.1	1.9	2.2	2.1

Friends left school (% with half or more)	Wave 1	6	7	7	7	8	8	4	8	6
	Wave 2				19	24	22	19	23	21

Religiosity (% not very/not at all religious)	Wave 1	72	64	68	71	65	68	71	63	67
	Wave 2				71	69	70	73	67	69

Values at waves 2 and 3 are weighted values

a Does not include pregnancy among those reporting sexual debut between waves 2 and 3.

**Table 2:** Sexual debut and expectation of tertiary education: univariate associations with other expectations, psychosocial factors and risk behaviours.

		Wave first reported	Sexual debut (*N* = 5061)				Expectation of tertiary education (*N* = 5061)				Participation in tertiary education (*N* = 2130)		
			Yes (wave 1)	Yes (wave 2)	No both waves	*p*	No (wave 1)	No (wave 2)	Yes both waves	*p*	No (Wave 3)	Yes (wave 3)	*p*
			Column%	Column%	Column%		Column%	Column%	Column%		Column%	Column%	
Pregnancy/partner pregnancy		Wave 2	17	6	N/A	***	5	8	3	***	7	3	**
		Wave 3	33	15	N/A	***	13	14	3	***	15	4	***

Early school leaving		Wave 2	49	31	14	***	35	36	13	***	47	15	***
		Wave 3	84	69	36	***	73	73	33	***	81	33	***

Expectations of early transition^a^	Expect job	Wave 1	45	43	37	***	50	46	33	***	46	33	
		Wave 2	24	19	13		21	25	11		24	12	
		Not reported either wave	31	37	50		30	28	56		30	54	
	Expect cohabitation	Wave 1	48	33	20	***	37	34	21	***	36	24	***
		Wave 2	10	12	4		8	8	7		10	6	
		Not reported either wave	41	55	76		56	58	72		54	70	
	Expect child	Wave 1	23	12	7	***	16	17	7	***	16	7	***
		Wave 2	5	4	1		3	4	1		2	1	
		Not reported either wave	72	84	92		80	79	92		82	91	

Other risk behaviours^b^	Regular cigarette smoking	Wave 1	42	14	4	***	17	16	8	***	18	10	***
		Wave 2	18	25	8		16	19	12		24	9	
		Not reported either wave	40	62	88		67	65	81		59	81	
	Regular drunkenness	Wave 1	50	17	6	***	21	21	10	***	19	11	***
		Wave 2	22	34	13		23	26	18		25	17	
		Not reported either wave	28	49	81		56	53	72		56	72	
	Regular cannabis use	Wave 1	11	3	1	***	3	6	2	***	4	2	*
		Wave 2	10	8	2		6	6	4		6	4	
		Not reported either wave	78	89	97		91	88	94		91	94	

Psychosocial factors^b^^c^	Not religious	Wave 1	74	73	63	***	75	72	61	***	72	65	***
		Wave 2	15	13	12		13	14	12		14	12	
		Not reported either wave	11	14	25		13	14	26		15	22	
	Low self-esteem	Wave 1	47	41	37	***	42	43	37	**	48	39	***
		Wave 2	9	11	12		10	10	13		12	12	
		Not reported either wave	45	48	50		48	46	50		40	49	
	Low parental monitoring	Wave 1	58	42	31	***	50	43	29	***	49	31	***
		Wave 2	15	14	9		12	14	10		13	11	
		Not reported either wave	28	44	60		39	42	61		38	58	
	Most friends left school	Wave 1	16	6	3	***	9	7	4	***	8	4	***
		Wave 2	37	28	10		26	26	13		30	14	
		Not reported either wave	46	66	86		66	67	83		62	82	
	High spending money	Wave 1	61	50	34	***	45	48	39	***	43	41	*
		Wave 2	23	20	16		19	20	17		22	16	
		Not reported either wave	16	30	50		36	32	44		35	43	
	Current boy/girlfriend	Wave 1	57	31	11	***	27	30	20	***	32	19	***
		Wave 2	20	35	12		20	21	18		24	18	
		Not reported either wave	23	34	77		53	48	62		44	63	

Note: Percentages are weighted values. N/A = Not applicable.

a Binary expectations contrast ‘very likely/likely’ with rest.

b Regular drunkenness denotes drunk once a week or more often, p values show results of Chi-square tests on univariate associations with outcome.

c Religiosity contrasts not/not at all religious; self esteem and parental monitoring contrast lowest tertile of scores with rest; friends left school contrasts responses including half or more with remainder; high spending money contrasts those in top 50% of scores with rest.

**Table 3:** Associations between early sexual debut and tertiary education: effects of adjusting for wave 2 academic achievement, social background and wave 1 covariates.

		No expectation of tertiary education (at wave 2) *N* = 5061						No participation in tertiary education (at wave 3) *N* = 2130						

		Stage 1			Stage 2			Stage 1			Stage 2			
		OR	95% CI	*p*	OR	95% CI	*p*	OR	95% CI	*p*	OR	95% CI	*p*	
Sexual debut	Wave 1	1.50	(1.14–1.99)	**	1.15	(0.81–1.64)		2.12	(1.47–3.05)	***	1.83	(1.17–2.86)	**	
	Wave 2	1.56	(1.24–1.97)	***	1.39	(1.08–1.80)	*	1.79	(1.30–2.48)	***	1.77	(1.17–2.67)	**	
	
Gender	Female	0.67	(0.56–0.81)	***	0.68	(0.56–0.82)	***	0.89	(0.69–1.14)	0.67		(0.49–0.90)	**	
	
*Wave 2 Academic achievement*														
Standard grade scores	Decile 9 & 10 (top scores)	1.00			1.00			1.00			1.00			
	Decile 8	2.12	(1.26–3.57)	***	1.71	(0.96–3.05)	*	3.16	(1.76–5.68)	***	2.89	(1.53–5.46)	**	
	Decile 7	2.67	(1.80–3.95)	***	1.89	(1.23–2.89)	**	5.08	(3.66–7.06)	***	4.52	(3.19–6.39)	***	
	Decile 6	4.44	(2.95–6.69)	***	2.82	(1.74–4.56)	***	8.39	(4.93–14.27)	***	6.93	(3.89–12.36)	***	
	Decile 5	6.79	(5.09–9.06)	***	4.13	(3.14–5.44)	***	12.18	(8.35–17.75)	***	9.98	(6.45–15.43)	***	
	Decile 4	12.70	(9.22–17.48)	***	6.38	(4.59–8.87)	***	17.44	(9.97–30.51)	***	14.89	(8.59–25.78)	***	
	Decile 3	10.65	(7.36–15.39)	***	5.38	(3.67–7.87)	***	17.84	(11.18–28.45)	***	13.73	(8.43–22.36)	***	
	Decile 2	15.93	(10.60–23.94)	***	7.72	(5.11–11.64)	***	20.87	(12.97–33.56)	***	14.39	(8.41–24.61)	***	
	Decile 1 (lowest scores)	18.62	(14.31–24.24)	***	8.05	(5.98–10.84)	***	28.64	(19.55–41.95)	***	19.67	(11.48–33.71)	***	
	
*Social background*														
Highest parental social class	Professional				1.00						1.00			
	Managerial/Technical				1.43	(0.94–2.17)					0.98	(0.57–1.68)		
	Skilled non-manual				1.53	(1.01–2.33)	*				1.09	(0.62–1.89)		
	Skilled manual				1.93	(1.22–3.05)	**				1.02	(0.56–1.87)		
	Partly skilled				1.67	(1.06–2.63)	*				1.86	(0.94–3.68)		
	Unskilled				1.25	(0.80–1.95)					0.96	(0.47–2.00)		
	
Highest parent educational level	Degree/advanced qualification				1.00						1.00			
	Attended college/university				1.08	(0.87–1.34)					1.31	(0.91–1.88)		
	Highers				1.53	(1.11–2.13)	*				0.68	(0.45–1.04)		
	Standard grades				1.45	(1.08–1.94)	*				1.35	(0.83–2.18)		
	At school post-16				1.69	(0.94–3.03)					1.12	(0.38–3.29)		
	Left school at 16				2.07	(1.45–2.94)	***				1.25	(0.80–1.97)		
	
Do not live with both biological parents					0.82	(0.67–1.00)					1.41	(1.14–1.75)	**	
	
Neighbourhood deprivation	DEPCAT 1 (most affluent)				1.00						1.00			
	DEPCAT 2				0.84	(0.64–1.11)					0.88	(0.63–1.24)		
	DEPCAT 3				0.81	(0.60–1.08)					1.17	(0.76–1.81)		
	DEPCAT 4				1.01	(0.68–1.51)					0.96	(0.62–1.49)		
	DEPCAT 5				1.13	(0.81–1.57)					0.99	(0.63–1.56)		
	DEPCAT 6				1.06	(0.64–1.74)					0.98	(0.64–1.50)		
	DEPCAT 7 (most deprived)				1.26	(0.72–2.21)					2.94	(1.22–7.08)		
	
School staying on rate from S4 to S5	Increasing %				0.73	(0.24–2.21)					1.25	(0.17–9.51)		
	
*Wave 1 school engagement*														
Like school	Strongly agree				1.00						1.00			
	Agree				1.54	(1.01–2.35)	*				1.28	(0.62–2.65)		
	Unsure				1.67	(1.04–2.67)	*				1.12	(0.50–2.51)		
	Disagree				1.55	(0.91–2.62)					1.35	(0.62–2.95)		
	Strongly disagree				1.40	(0.90–2.17)					1.16	(0.42–3.26)		
	
Skip school	Strongly disagree				1.00						1.00			
	Disagree				0.96	(0.76–1.22)					1.01	(0.78–1.30)		
	Unsure				1.07	(0.84–1.36)					0.77	(0.54–1.10)		
	Agree				1.01	(0.75–1.36)					0.70	(0.38–1.30)		
	Strongly agree				0.68	(0.36–1.29)					0.33	(0.13–0.83)		
	
*Wave 1 expectations for age 18*														
Expect further education	Very likely				1.00						1.00			
	Likely				1.38	(1.11–1.73)	**				1.13	(0.79–1.62)		
	Unsure				2.78	(2.09–3.70)	***				1.93	(1.30–2.86)	**	
	Unlikely				2.75	(1.91–3.95)	***				2.59	(1.48–4.52)	**	
	Very unlikely				3.14	(1.88–5.24)	***				1.79	(0.83–3.84)		
	
Expect job	Very unlikely				1.00						1.00			
	Unlikely				1.63	(0.92–2.88)					0.75	(0.37–1.52)		
	Unsure				1.60	(0.85–3.03)					0.68	(0.35–1.32)		
	Likely				1.62	(0.87–3.01)					0.68	(0.33–1.37)		
	Very likely				1.90	(1.00–3.61)	*				0.66	(0.30–1.48)		
	
Expect cohabit	Very unlikely				1.00						1.00			
	Unlikely				0.84	(0.61–1.16)					1.59	(0.96–2.64)		
	Unsure				0.80	(0.56–1.14)					1.45	(0.79–2.66)		
	Likely				0.93	(0.60–1.45)					1.11	(0.58–2.11)		
	Very likely				0.99	(0.59–1.66)					1.48	(0.73–3.02)		
	
Expect child	Very unlikely				1.00						1.00			
	Unlikely				1.10	(0.91–1.32)					1.10	(0.73–1.64)		
	Unsure				1.22	(0.97–1.54)					1.01	(0.62–1.65)		
	Likely				1.20	(0.89–1.62)					1.28	(0.76–2.15)		
	Very likely				1.88	(1.07–3.30)	*				1.23	(0.47–3.22)		
	
*Wave 1 psychosocial factors*														
Self esteem	Decreasing				1.19	(1.01–1.41)	*				1.37	(1.03–1.83)	*	
	
Religious belief	Religious				1.00						1.00			
	Unsure				1.31	(0.87–1.96)					1.45	(0.90–2.33)		
	Not religious				1.30	(0.88–1.92)					1.32	(0.80–2.17)		
	Not at all religious				1.60	(1.12–2.29)	**				1.27	(0.74–2.16)		
	
Parental monitoring	Decreasing				1.11	(0.96–1.28)					1.32	(1.12–1.55)	**	
	
Friends left school	None				1.00						1.00			
	A few				1.11	(0.92–1.34)					1.32	(1.04–1.68)	*	
	Half				1.33	(0.84–2.10)					1.41	(0.70–2.85)		
	Most				0.96	(0.56–1.64)					1.29	(0.47–3.56)		
	All				0.10	(0.01–1.68)					2.60	(0.16–43.04)		
	
Spending money	Increasing				1.00	(0.99–1.01)					0.99	(0.98–1.00)		
	
Current boy/girlfriend	yes				0.95	(0.76–1.17)					1.35	(1.02–1.80)	*	
	
*Wave 1 risk behaviours*														
Cigarette use	Never tried				1.00						1.00			
	Tried				1.12	(0.94–1.32)					0.97	(0.70–1.35)		
	Use occasionally				1.20	(0.86–1.67)					1.29	(0.88–1.89)		
	Use regularly				1.26	(0.91–1.73)					1.16	(0.64–2.10)		
	
Drunkenness	Never drunk				1.00						1.00			
	Drunk once/twice a year				1.27	(1.06–1.52)	*				0.92	(0.68–1.23)		
	Drunk about once a month				1.13	(0.86–1.47)					1.02	(0.71–1.47)		
	Drunk about once a week				1.44	(1.08–1.91)	*				0.94	(0.57–1.56)		
	Drunk more than once a week				0.93	(0.59–1.46)					2.28	(0.99–5.28)		
	
Cannabis use	Never tried				1.00						1.00			
	Tried				0.98	(0.74–1.30)					0.82	(0.58–1.17)		
	Use occasionally				0.80	(0.49–1.32)					0.73	(0.39–1.35)		
	Use regularly				1.15	(0.64–2.04)					0.69	(0.30–1.59)		

Note: All models adjust for arm of trial and age at wave 2. *p < 0.05,**p < 0.01, ***p < 0.001.

**Table 4:** Effect of sexual debut on tertiary education after adjusting for change.

			Timing of sexual debut	No expectation of tertiary education (at wave 2) *N* = 5061			No participation in tertiary education (at wave 3) *N* = 2130		
				OR	95% CI	*p*	OR	95% CI	*p*
Model adjusted for controls in Stage 2 (Table 3)			Wave 1	1.15	(0.81–1.64)		1.83	(1.17–2.86)	**
			Wave 2	1.39	(1.08–1.80)	*	1.77	(1.17–2.67)	**

Stage 3	Model number								
Adjusting for events	1	Adjustment for pregnancy	Wave 1	1.10	(0.76–1.58)		1.67	(1.11–2.52)	*
			Wave 2	1.37	(1.06–1.77)	*	1.72	(1.12–2.65)	*
	2	Model 1 plus adjustment for early leaving	Wave 1	1.05	(0.74–1.50)		1.36	(0.90–2.07)	
			Wave 2	1.34	(1.04–1.72)	*	1.35	(0.89–2.07)	

Adjusting for changed expectations of early transition	3	Model 1 plus adjustment for w2 expectation of job, cohabitation and childbearing	Wave 1	0.99	(0.68–1.44)		1.64	(1.07–2.52)	*
			Wave 2	1.30	(1.01–1.67)	*	1.61	(1.08–2.42)	*
	3a	Model 3 plus adjustment for w2 expectation of no tertiary education	Wave 1	N/A			1.63	(1.04–2.56)	*
			Wave 2	N/A			1.51	(1.00–2.26)	*

Adjusting for psychosocial changes	4	Model 1 plus adjustment for w2 current boy/girlfriend and spending money	Wave 1	0.98	(0.68–1.43)		1.53	(1.01–2.33)	*
			Wave 2	1.28	(0.97–1.68)		1.51	(0.99–2.29)	
	5	Model 1 plus adjustment for w2 parental monitoring, friends left school, self-esteem, religiosity and substance use	Wave 1	0.99	(0.69–1.42)		1.65	(1.16–2.35)	**
			Wave 2	1.28	(0.95–1.71)		1.59	(1.04–2.45)	*
	6	Model 1 plus combined adjustments in 4 and 5	Wave 1	0.92	(0.64–1.32)		1.54	(1.07–2.22)	*
			Wave 2	1.22	(0.90–1.65)		1.42	(0.93–2.16)	

Note: *p < 0.05,**p < 0.01, ***p < 0.001.

## Footnotes

1 Incentives at wave 3 were (1) none (2) single large prize of £500 (3) single prize offered for each school (4) definite reward (£10) on completion of questionnaire.

2 The level of placing requests (ie requests for a pupil to attend a secondary school different from the one allocated on the basis of catchment area) is likely to reflect a school's academic performance.

3 DEPCAT scores for Scottish postcode sectors ranging from 1 (most affluent) to 7 (most deprived) were calculated from Carstairs scores based on a combination of four variables derived from 2001 census small area statistics relating to overcrowding, male unemployment, low social class and car ownership. See Philip McLoone, *Carstair Scores for Scottish Postcode Sectors from the 2001 Census*, MRC Social and Public Health Sciences Unit, March 2004 http://www.msoc-mrc.gla.ac.uk/Publications/pub/PDFs/PHRU/Carstairs.pdf.

4 Computer Assisted Standard Occupational Coding (CASOC) was used.

5 Scottish government statistics.
